# Emergence of Acquired Dolutegravir Resistance in Treatment-experienced People With HIV in Lesotho

**DOI:** 10.1093/cid/ciae185

**Published:** 2024-04-03

**Authors:** Nadine Tschumi, Blaise Lukau, Katleho Tlali, Lipontso Motaboli, Mpho Kao, Mathebe Kopo, Kathrin Haenggi, Moleboheng Mokebe, Klaudia Naegele, Irene Ayakaka, Karoline Leuzinger, Jennifer A Brown, Niklaus D Labhardt

**Affiliations:** Division of Clinical Epidemiology, Department of Clinical Research, University Hospital Basel, Basel, Switzerland; Department of Clinical Research, University of Basel, Basel, Switzerland; SolidarMed, Partnerships for Health, Maseru, Lesotho; SolidarMed, Partnerships for Health, Maseru, Lesotho; SolidarMed, Partnerships for Health, Maseru, Lesotho; SolidarMed, Partnerships for Health, Maseru, Lesotho; SolidarMed, Partnerships for Health, Maseru, Lesotho; Division of Clinical Epidemiology, Department of Clinical Research, University Hospital Basel, Basel, Switzerland; Department of Clinical Research, University of Basel, Basel, Switzerland; SolidarMed, Partnerships for Health, Maseru, Lesotho; Clinical Virology, Laboratory Medicine, University Hospital Basel, Switzerland; SolidarMed, Partnerships for Health, Maseru, Lesotho; Clinical Virology, Laboratory Medicine, University Hospital Basel, Switzerland; Division of Clinical Epidemiology, Department of Clinical Research, University Hospital Basel, Basel, Switzerland; Department of Clinical Research, University of Basel, Basel, Switzerland; Division of Clinical Epidemiology, Department of Clinical Research, University Hospital Basel, Basel, Switzerland; Department of Clinical Research, University of Basel, Basel, Switzerland

**Keywords:** dolutegravir, HIV, antiretroviral therapy, drug resistance, Africa

## Abstract

**Background:**

Since 2019, the World Health Organization has recommended dolutegravir-based antiretroviral therapy (ART) as the preferred regimen for human immunodeficiency virus management. Large-scale programmatic transitioning to dolutegravir-based ART was subsequently implemented across Africa, often in the absence of recent viral load testing and without access to genotypic resistance testing (GRT) in case of viremia.

**Methods:**

This study assessed emerging dolutegravir resistance in the routine care of the Viral Load Cohort North-East Lesotho. We included pediatric and adult participants who changed from nonnucleoside reverse transcriptase inhibitor– to dolutegravir-based ART and had at least 1 viral load assessment before and after the change. We sequenced available samples of participants fulfilling the additional virological criteria of having 2 viremic episodes while taking dolutegravir, with at least 1 viral load ≥500 copies/mL taken ≥18 months after changing to dolutegravir.

**Results:**

Among 15 349 participants, 157 (1.0%) met the virological criteria, and GRT was successful for 85 (0.6%). Among these 85, 8 (9.4%) had dolutegravir resistance, with 2 (2.4%) and 6 (7.1%) predicted to have intermediate- and high-level dolutegravir resistance, respectively. One participant had 2, 2 had 1, and 5 had 0 active drugs in their regimen. A GRT from before the change to dolutegravir was available for 5 of these 8 participants: 4 had 0 and 1 had 1 active drug in their nonnucleoside transcriptase inhibitor–based regimen.

**Conclusions:**

Nine percent of people with persistent or recurring human immunodeficiency virus viremia ≥18 months after changing to dolutegravir-based ART had dolutegravir resistance. Detection and management of emerging dolutegravir resistance must be addressed across Africa.

## Teaser

In a cohort study in Lesotho, the prevalence of dolutegravir resistance was 9.4% among people with persistent or recurring HIV viremia ≥18 months after changing to dolutegravir-based antiretroviral therapy. Better measures are needed for early detection of dolutegravir resistance.

## Graphical Abstract

**Graphical Abstract:**
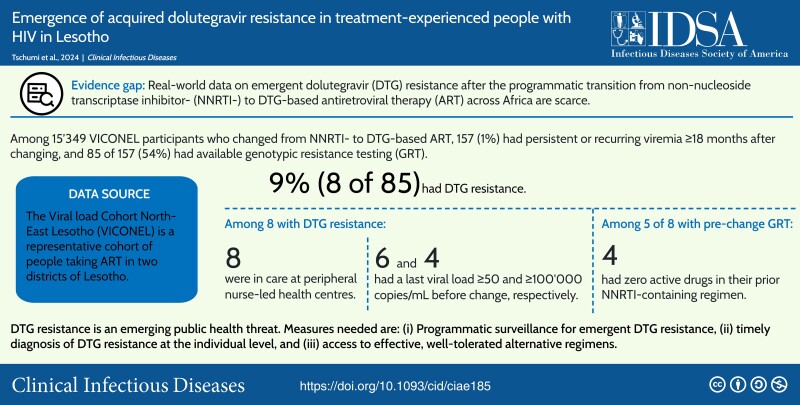
This graphical abstract is also available at Tidbit: https://tidbitapp.io/tidbits/emergence-of-acquired-dolutegravir-resistance-in-treatment-experienced-people-with-hiv-in-lesotho-f28c546c-c7e2-43e6-8dcc-736fc4da4763?utm_campaign=tidbitlinkshare&utm_source=ITP

Dolutegravir is a second-generation human immunodeficiency virus (HIV) integrase strand transfer inhibitor (INSTI) with excellent efficacy, potency, tolerability, and a higher genetic barrier to resistance than previously used antiretroviral therapy (ART) regimens [1–5]. These characteristics of dolutegravir, along with its availability at low cost [6] and rising levels of nonnucleoside reverse transcriptase inhibitor (NNRTI) resistance [7], prompted the World Health Organization to recommend dolutegravir alongside 2 nucleoside reverse transcriptase inhibitors (NRTIs) as the preferred treatment option for most people with HIV [8]. As of 2022, 91% of all adults with HIV in low- and middle-income countries were receiving dolutegravir-based ART [6]. Large-scale programmatic transitioning from previous NNRTI-based to dolutegravir-based ART was implemented pragmatically, often in the absence of recent viral load testing and without access to genotypic resistance testing (GRT) in case of viremia [9].

Real-world viral load outcomes reported from routine care settings of treatment-experienced individuals after programmatic transitioning to dolutegravir are encouraging, and virological failure has become uncommon with dolutegravir-based regimens [10–12]. Dolutegravir resistance is thus far considered rare, capacity for GRT is extremely limited in many settings, and treatment guidelines in southern Africa state that resistance testing is generally not necessary within the first 24 months of initiating dolutegravir [13–15]. Consequently, the majority of people taking dolutegravir-based ART do not routinely have access to GRT in case of virological failure, and data on dolutegravir resistance in Africa are scarce. Two randomized trials evaluated dolutegravir for second-line ART in people with virological failure while taking NNRTI-containing first-line therapy [16, 17]. In the NADIA trial, 235 participants were assigned to receive dolutegravir; among 24 with sustained viremia at 96 weeks, 9 (37.5%) had developed dolutegravir resistance [16]. In the DAWNING trial, 312 participants were assigned to receive dolutegravir; among 11 with sustained viremia by 52 weeks, 2 (18.2%) had developed dolutegravir resistance [17]. Both trials indicate that prior NRTI backbone resistance is associated with acquisition of dolutegravir resistance. Four observational studies within national HIV programs in Malawi, Nigeria, and Tanzania reported a total of 19 cases of INSTI resistance-associated mutations. These typically occurred in conjunction with NRTI backbone resistance; however, in most cases resistance data before dolutegravir exposure were not available [11, 18–20]. A recent global collaborative cohort analysis reported 3% intermediate-level and 1% high-level resistance to dolutegravir among 599 individuals with viremia but included only 9 samples from Africa. Given the high prevalence of NRTI resistance in people with treatment failure taking NNRTI-based regimens [21], pragmatic transitioning might have facilitated the emergence of acquired dolutegravir resistance [22].

Here, we report on evolving resistance patterns before and after the treatment change among individuals in routine care with persistent or recurring viremia and available GRT beyond 18 months after changing from NNRTI- to dolutegravir-based ART in Lesotho, southern Africa.

## METHODS

### Study Design

The Viral Load Cohort North-East Lesotho (VICONEL) is a prospective open cohort established in 2016. VICONEL includes all people with HIV who receive routine HIV viral load testing at 21 health centers and 3 hospitals in Butha-Buthe and Mokhotlong districts, Lesotho. The cohort setup has been described previously [23]. The current study assesses the emergence of dolutegravir resistance after changing from NNRTI- to dolutegravir-based ART. Reporting follows the STROBE statement and the checklist for studies of HIV drug resistance prevalence or incidence [24, 25].

### Setting

Lesotho has an adult HIV prevalence of 19.3% [26]. ART provision is mostly nurse-led. In 2019, Lesotho national guidelines were amended to recommend dolutegravir-based ART as the preferred first- and second-line regimen. The major rollout of dolutegravir took place in 2020. After initially requiring documented viral suppression before the regimen change, people without a documented viral load or with an unsuppressed viral load were later also changed to dolutegravir [9]. Dolutegravir was initially only available as a fixed-dose combination for people weighing at least 35 kg and as a nonfixed-dose combination for people weighing at least 20 kg; treatment with lopinavir-based ART was initially recommended for children below 20 kg.

Although current national ART guidelines foresee GRT following sustained viremia of ≥1000 copies/mL despite enhanced adherence counseling [15], this requires approval by both a district and a national ART advisory committee. Laboratory capacities are lacking, necessitating sample transfer to South Africa and funding is limited. Because of these operational and cost barriers, GRT is extremely rare in this setting.

### Participants

This study includes VICONEL participants who fulfilled the following inclusion criteria: Written informed consent for further use of biobanked samples; changed from ART containing an NNRTI (efavirenz or nevirapine) alongside 2 NRTIs (abacavir [ABC], zidovudine, or tenofovir disoproxil fumarate [TDF] together with lamivudine [3TC]) to ART containing dolutegravir plus 2 NRTIs before 19 October 2021; with at least 1 viral load available before and after change. No age restrictions were applied. Samples were sequenced for participants fulfilling the additional virological criteria of having at least 1 viral load ≥50 copies/mL measured ≥90 days after changing to dolutegravir and at least 1 further viral load ≥500 copies/mL taken ≥18 months after changing to dolutegravir. For each participant fulfilling virological criteria, the last available sample with a viral load ≥500 copies/mL while taking dolutegravir was sequenced (technical cutoff for GRT). If dolutegravir resistance-associated mutations were observed, we additionally sequenced all stored prior samples with a viral load ≥500 copies/mL while taking dolutegravir. Finally, we sequenced the last available sample before the change to dolutegravir if ≥500 copies/mL and it was taken ≤18 months before the change.

### Data Sources/Measurements

Demographic, clinical, and treatment-related data were derived from the VICONEL database.

For routine viral load testing, blood samples collected at participating clinics are transported to the Butha-Buthe district hospital for processing. Leftover sample volume is stored at −80°C. For near full-genome sequencing, samples were shipped on dry ice to the Virology Laboratory of the University Hospital Basel in Switzerland. HIV RNA genomes were extracted from plasma samples with the QIAmp Blood Kit (Qiagen, Hilden, Germany) according to the manufacturer's instruction. Primers were designed for an HIV-1 subtype C reference genome generated through alignment and merging of publicly available HIV-1 subtype C sequences. Polymerase chain reaction was conducted for amplicons around 500–1000 bp in length using the Iproof High Fidelity DNA Polymerase kit (BioRad, CA, USA). A negative and a positive control were included in each run. Libraries were prepared using the Nextera XT DNA Library Preparation Kit (Illumina, CA, USA) following the manufacturers’ instructions. Next-generation sequencing was performed on a MiSeq platform (Illumina). Bioinformatic analyses including Illumina sequencing read quality control, adapter trimming, mapping, and alignment were done with the commercially available SMARTGene next-generation sequencing HIV-1 software (SMARTGene, Lausanne, Switzerland). Resistance-associated mutation calling was done with a variant frequency threshold of 5% to facilitate the early detection of HIV resistance-associated mutations while maintaining detection specificity [27]. We used the Stanford HIV drug resistance database (version 9.5.0) to interpret resistance-associated mutations [28]. Samples were excluded if coverage was below 1 read at any INSTI resistance-associated mutation position. Samples with coverage below 10 reads at any resistance-associated mutation position in the reverse transcriptase, protease, or integrase region were flagged as such. Using consensus sequences, we obtained resistance-associated mutations as well as resistance levels (high-level resistance, intermediate resistance, low-level resistance, potential low-level resistance, and susceptible) for each of the 3 drugs of each participant's ART regimen at each sample time point through the Stanford HIV drug resistance database [28].

Data closure for initial sample selection, selection of additional samples of participants with detected dolutegravir resistance, and analysis were on 20 April 2023, 15 September 2023, and 1 February 2024, respectively.

### Variables/Statistical Methods

Based on the resistance levels for each drug in each participants’ ART regimen at each time point, we calculated specific susceptibility scores. For each drug, high-level resistance, intermediate resistance, low-level resistance, potential low-level resistance, and susceptible were assigned values of 0.00, 0.25, 0.50, 0.75, and 1.00, respectively, as has been described elsewhere [29]. Dolutegravir and NNRTI susceptibility scores were considered separately (possible range: 0.00–1.00), and the backbone susceptibility score was defined as the sum of both NRTI susceptibility scores (possible range: 0.00–2.00). Lower susceptibility scores indicate lower susceptibility (higher resistance). We consider dolutegravir resistance to be present when susceptibility is ≤0.50 (ie, at least low-level resistance). Accordingly, we consider a drug to be active when susceptibility is >0.50.

All variables are summarized as frequencies and percentages for categorical variables and medians and interquartile ranges (IQRs) for continuous variables. Comparisons were made using Fisher tests for categorical variables and Wilcoxon rank sum test of medians for continuous variables. All analyses were done using R version 4.3.1 (16.06.2023).

### Ethical Considerations

The VICONEL cohort, within which this study was conducted, has been approved by the National Health Research Ethics Committee in Lesotho (ID134-2016, last renewal 16 May 2023). Informed consent was waived for the reporting of routine data. Written informed consent for the further use of biobanked samples was provided by all adult participants (≥18 years) and caregivers of participating minors for whom resistance data are reported.

## RESULTS

### Study Population

**Figure 1.**
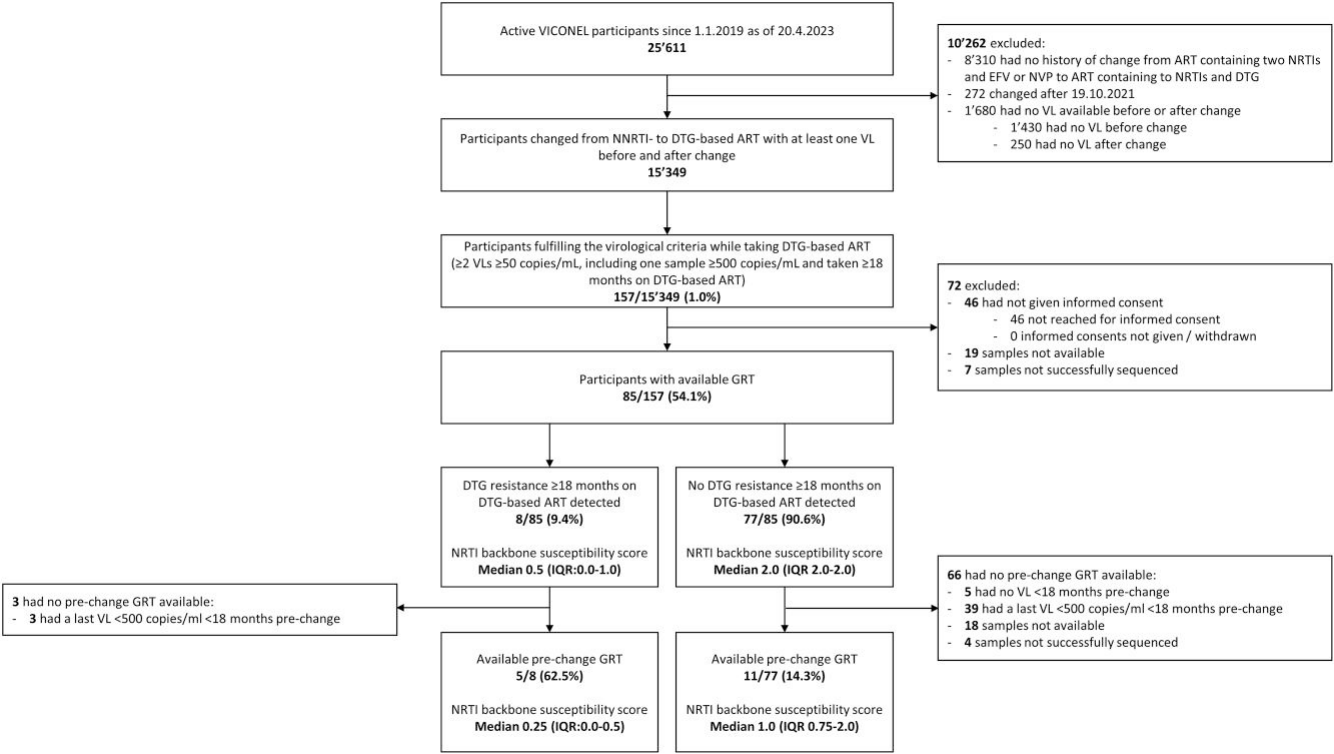
Study flow chart. ART, antiretroviral therapy; DTG, dolutegravir; EFV, efavirenz; GRT, genotypic resistance test; IQR, interquartile range; NNRTI, nonnucleoside reverse transcriptase inhibitor; NRTI, nucleoside/nucleotide reverse transcriptase inhibitor; NVP, nevirapine; VL: viral load; VICONEL, Viral Load Cohort North-East Lesotho.

A total of 15 349 VICONEL participants changed from NNRTI- to dolutegravir-based ART ≥18 months before data closure (Figure 1, Table 1). Suppression rates and correlates of viremia after change to dolutegravir among pediatric [30] and adult [12] VICONEL participants have been described elsewhere. Virological criteria for the present analysis, namely having a viral load ≥500 copies/mL ≥18 months and at least 1 further viral load ≥50 copies/mL ≥90 days after changing to dolutegravir, were fulfilled by 157/15 349 (1.0%). Table 1 displays participants’ characteristics, both overall and stratified by fulfilling vs not fulfilling the virological criteria. Of the 157 fulfilling the virological criteria, informed consent, a sample for GRT, and a GRT result were available for 85 (54.1%) (Figure 1, Table 2).

**Table 1.** Participants’ Characteristics

		Overall	Not Fulfilling Virological Criteria	Fulfilling Virological Criteria	*P*
n		15 349	15 192	157	
Sex (%)	Female	9936 (64.7)	9852 (64.8)	84 (53.5)	.004
	Male	5413 (35.3)	5340 (35.2)	73 (46.5)	
Age in years at change (median [IQR])	…	43.2 [34.7–53.9]	43.4 [34.8–53.9]	29.2 [14.7–42.1]	<.001
(%)	<12	239 (1.6)	215 (1.4)	24 (15.3)	<.001
	12–17	478 (3.1)	444 (2.9)	34 (21.7)	
	18–24	584 (3.8)	570 (3.8)	14 (8.9)	
	25–44	7094 (46.2)	7036 (46.3)	58 (36.9)	
	45–64	5827 (38.0)	5806 (38.2)	21 (13.4)	
	≥65	1126 (7.3)	1120 (7.4)	6 (3.8)	
	Missing	1 (0.0)	1 (0.0)	0 (0.0)	
Years taking ART before change to DTG (median [IQR])	…	5.5 [3.5–9.0]	5.5 [3.5–9.0]	5.7 [3.3–8.5]	.446
(%)	<5	6806 (44.3)	6734 (44.3)	72 (45.9)	.749
	5–10	5846 (38.1)	5785 (38.1)	61 (38.9)	
	>10	2697 (17.6)	2673 (17.6)	24 (15.3)	
Facility type (%)	Health center	8985 (58.5)	8903 (58.6)	82 (52.2)	.126
	Hospital	6364 (41.5)	6289 (41.4)	75 (47.8)	
District (%)	Butha-Buthe	9472 (61.7)	9377 (61.7)	95 (60.5)	.819
	Mokhotlong	5877 (38.3)	5815 (38.3)	62 (39.5)	
Last viral load ≤1 mo before change (%)	<50 copies/mL	3382 (22.0)	3361 (22.1)	21 (13.4)	<.001
	50–499 copies/mL	259 (1.7)	253 (1.7)	6 (3.8)	
	500–999 copies/mL	44 (0.3)	41 (0.3)	3 (1.9)	
	1000–9999 copies/mL	61 (0.4)	59 (0.4)	2 (1.3)	
	10 000–99 999 copies/mL	41 (0.3)	38 (0.3)	3 (1.9)	
	≥100 000 copies/mL	21 (0.1)	20 (0.1)	1 (0.6)	
	missing	11 541 (75.2)	11 420 (75.2)	121 (77.1)	
Last viral load ≤18 mo before change (%)	<50 copies/mL	11 641 (75.8)	11 572 (76.2)	69 (43.9)	<.001
	50–499 copies/mL	1649 (10.7)	1629 (10.7)	20 (12.7)	
	500–999 copies/mL	266 (1.7)	258 (1.7)	8 (5.1)	
	1000–9999 copies/mL	371 (2.4)	352 (2.3)	19 (12.1)	
	10 000–99 999 copies/mL	242 (1.6)	223 (1.5)	19 (12.1)	
	≥100 000 copies/mL	78 (0.5)	68 (0.4)	10 (6.4)	
	Missing	1102 (7.2)	1090 (7.2)	12 (7.6)	
Months between last viral load while taking NNRTI and change to DTG (median [IQR])		3.9 [0.9–8.1]	3.9 [0.9–8.1]	4.0 [1.0–7.7]	.684
Last viral load ≥18 mo after change to DTG (%)	<50 copies/mL	13 638 (88.9)	13 557 (89.2)	81 (51.6)	<.001
	50–499 copies/mL	383 (2.5)	360 (2.4)	23 (14.6)	
	500–999 copies/mL	33 (0.2)	28 (0.2)	5 (3.2)	
	1000–9999 copies/mL	68 (0.4)	54 (0.4)	14 (8.9)	
	10 000–99 999 copies/mL	75 (0.5)	50 (0.3)	25 (15.9)	
	≥100 000 copies/mL	27 (0.2)	18 (0.1)	9 (5.7)	
	missing	1125 (7.3)	1125 (7.4)	0 (0.0)	
Participant reports at least 1 missed dose at last viral load ≥18 mo after change (%)	No	13 756 (89.6)	13 612 (89.6)	144 (91.7)	<.001
	Yes	279 (1.8)	269 (1.8)	10 (6.4)	
	Missing	1314 (8.6)	1311 (8.6)	3 (1.9)	
Months between change to DTG and last viral load (median [IQR])		37.5 [32.9–41.7]	37.4 [32.9–41.6]	40.6 [35.9–44.8]	<.001
NNRTI before change to DTG (%)	EFV	13 878 (90.4)	13 753 (90.5)	125 (79.6)	<.001
	NVP	1471 (9.6)	1439 (9.5)	32 (20.4)	
NRTI backbone change at change to DTG (%)	new backbone	3090 (20.1)	3021 (19.9)	69 (43.9)	<.001
	No change	12 259 (79.9)	12 171 (80.1)	88 (56.1)	
Previous documented exposure to RAL^a^ (%)	No	15 349 (100.0)	15 192 (100.0)	157 (100.0)	NA

Stratified by fulfilling versus not fulfilling virological criteria for GRT after change to dolutegravir. *P* values: Fisher exact test for categorical, Wilcoxon rank sum test for continuous data.

Abbreviations: ART, antiretroviral therapy; DTG, dolutegravir; EFV, efavirenz; NA, not available; NNRTI, nonnucleoside reverse transcriptase inhibitor; NRTI, nucleoside reverse transcriptase inhibitor; NVP, nevirapine, RAL, raltegravir.

^a^Since 2016.

**Table 2.** Characteristics of the 85 Participants With Available Genotypic Resistance Testing Data

		Overall	No DTG Resistance	With DTG Resistance	*P*
n	…	85	77	8	
Sex (%)	F	47 (55.3)	42 (54.5)	5 (62.5)	.954
	M	38 (44.7)	35 (45.5)	3 (37.5)	
Age in years at change (median [IQR])	…	26.5 [15.3–38.5]	25.3 [15.3–38.5]	27.0 [21.6–36.2]	.869
(%)	<12	12 (14.1)	10 (13.0)	2 (25.0)	.415
	12–17	17 (20.0)	17 (22.1)	0 (0.0)	
	18–24	10 (11.8)	10 (13.0)	0 (0.0)	
	25–44	34 (40.0)	29 (37.7)	5 (62.5)	
	45–64	9 (10.6)	8 (10.4)	1 (12.5)	
	≥65	3 (3.5)	3 (3.9)	0 (0.0)	
Years taking ART before change to DTG (median [IQR]) (%)	…	6.0 [3.1–8.0]	5.7 [3.0–8.0]	6.4 [6.1–9.0]	.359
	<5	37 (43.5)	36 (46.8)	1 (12.5)	.108
	5–10	35 (41.2)	29 (37.7)	6 (75.0)	
	>10	13 (15.3)	12 (15.6)	1 (12.5)	
Facility type (%)	Health center	51 (60.0)	43 (55.8)	8 (100.0)	.041
	Hospital	34 (40.0)	34 (44.2)	0 (0.0)	
District (%)	Butha-Buthe	39 (45.9)	38 (49.4)	1 (12.5)	.106
	Mokhotlong	46 (54.1)	39 (50.6)	7 (87.5)	
Last viral load ≤1 mo before change (%)	<50 copies/mL	11 (12.9)	11 (14.3)	0 (.0)	.468
	50–499 copies/mL	2 (2.4)	1 (1.3)	1 (12.5)	
	500–999 copies/mL	1 (1.2)	1 (1.3)	0 (.0)	
	1000–9999 copies/mL	1 (1.2)	1 (1.3)	0 (0.0)	
	10 000–99 999 copies/mL	2 (2.4)	2 (2.6)	0 (0.0)	
	≥100 000 copies/mL	1 (1.2)	1 (1.3)	0 (0.0)	
	Missing	67 (78.8)	60 (77.9)	7 (87.5)	
Last viral load ≤18 mo before change to DTG (%)	<50 copies/mL	32 (37.6)	30 (39.0)	2 (25.0)	.005
	50–499 copies/mL	10 (11.8)	9 (11.7)	1 (12.5)	
	500–999 copies/mL	5 (5.9)	5 (6.5)	0 (0.0)	
	1000–9999 copies/mL	14 (16.5)	14 (18.2)	0 (0.0)	
	10 000–99 999 copies/mL	11 (12.9)	10 (13.0)	1 (12.5)	
	≥100 000 copies/mL	8 (9.4)	4 (5.2)	4 (50.0)	
	Missing	5 (5.9)	5 (6.5)	0 (0.0)	
Months between last viral load while taking NNRTI and change to DTG (median [IQR])		4.4 [1.2–7.7]	4.4 [1.2–7.6]	4.6 [1.4–8.3]	.784
NNRTI before change (%)	EFV	66 (77.6)	60 (77.9)	6 (75.0)	1
	NVP	19 (22.4)	17 (22.1)	2 (25.0)	
NRTI backbone change at change to DTG (%)	New backbone	36 (42.4)	31 (40.3)	5 (62.5)	.403
	No change	49 (57.6)	46 (59.7)	3 (37.5)	
Treatment regimen at last GRT (%)	ABC-3TC-DTG	18 (21.2)	16 (20.8)	2 (25.0)	.165
	AZT-3TC-DTG	7 (8.2)	5 (6.5)	2 (25.0)	
	TDF-3TC-DTG	60 (70.6)	56 (72.7)	4 (50.0)	
Viral load at last GRT (median)	500–999 copies/mL	7 (8.2)	7 (9.1)	0 (.0)	…
	1000–9999 copies/mL	33 (38.8)	29 (37.7)	4 (50.0)	
	10 000–99 999 copies/mL	33 (38.8)	30 (39.0)	3 (37.5)	
	≥100 000 copies/mL	11 (12.9)	11 (14.3)	0 (0.0)	
	missing	1 (1.2)	0 (0.0)	1 (12.5)	
Months between change to DTG and last GRT (median [IQR])		29.4 [24.0–34.6]	29.4 [23.8–34.0]	33.7 [28.5–38.2]	.086
Participant reports at least one missed dose at last GRT (%)	No	78 (91.8)	71 (92.2)	7 (87.5)	.48
	Yes	3 (3.5)	3 (3.9)	0 (0.0)	
	Missing	4 (4.7)	3 (3.9)	1 (12.5)	
Further unsuppressed viral load for the definition of virologic failure (in addition to the viral load ≥500 copies/mL taken ≥18 m after change to DTG) (%)	50–499 copies/mL	38 (44.7)	36 (46.8)	2 (25.0)	…
	500–999 copies/mL	6 (7.1)	5 (6.5)	1 (12.5)	
	1000–9999 copies/mL	20 (23.5)	18 (23.4)	2 (25.0)	
	10 000–99 999 copies/mL	17 (20.0)	14 (18.2)	3 (37.5)	
	≥100 000 copies/mL	4 (4.7)	4 (5.2)	0 (0.0)	

*P* values: Fisher exact test for categorical, Wilcoxon rank sum test for continuous data.

Abbreviations: ART, antiretroviral therapy; DTG, dolutegravir; EFV, efavirenz; GRT, genotypic resistance testing; NNRTI, nonnucleoside reverse transcriptase inhibitor; NRTI, nucleoside reverse transcriptase inhibitor; NVP, nevirapine.

### Detected Resistance Before Changing to Dolutegravir

**Figure 2.**
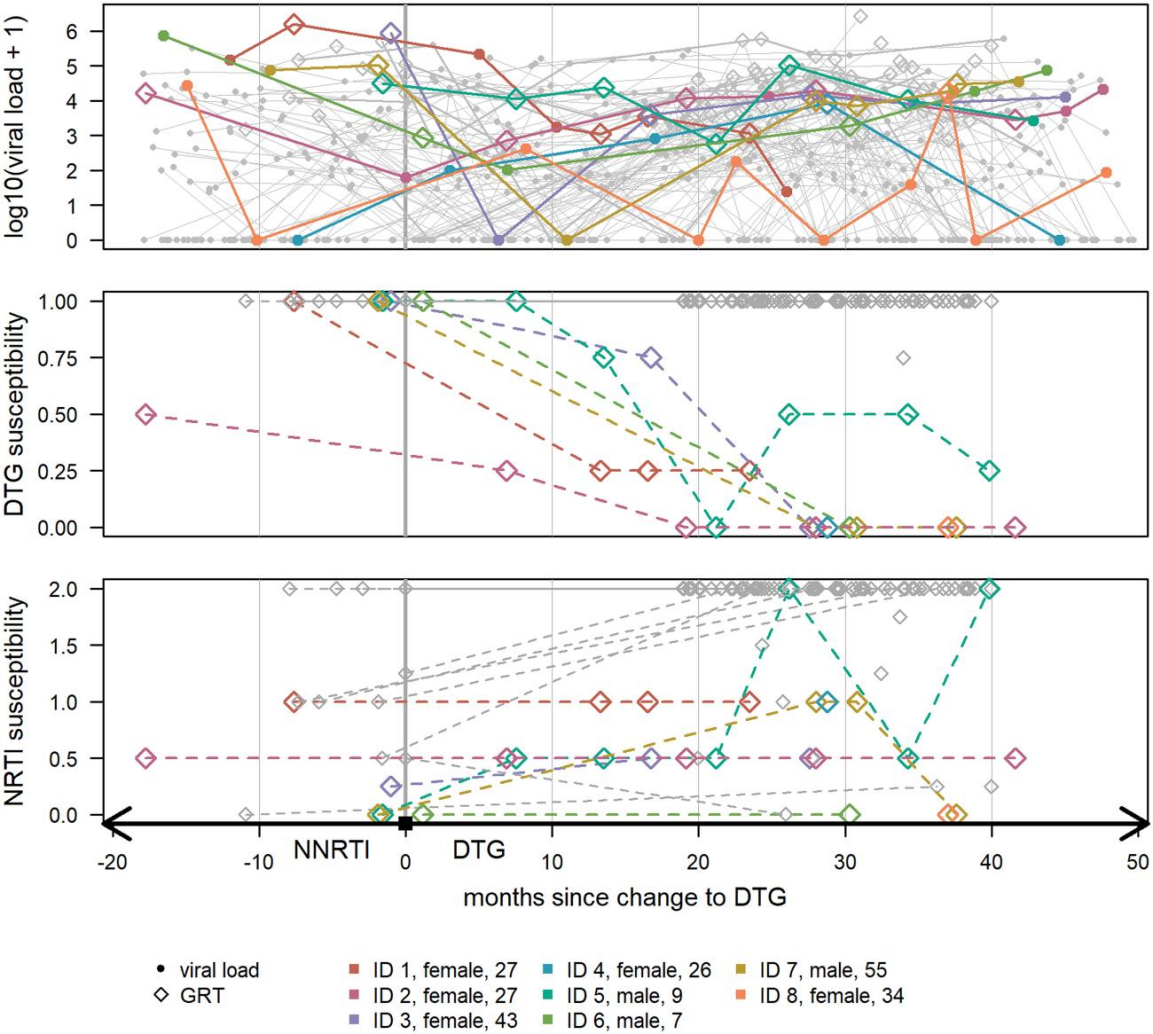
Viral load, dolutegravir, and NRTI backbone susceptibility from ≤18 mo pretransition until the last available, successfully sequenced sample. Susceptibility scores refer to the regimen at sampling. Age refers to the age in years at change to dolutegravir. DTG, dolutegravir; GRT, genotypic resistance test; NRTI, nucleoside/nucleotide reverse transcriptase inhibitor.

Of the 85 participants, 38 (44.7%) had a last viral load of ≥500 copies/mL in the past 18 months before changing to dolutegravir. In 16/38 (42.1%) a sample from this prechange viral load was available and GRT successful (Figure 1). Of these, 12/16 (75.0%) had NRTI and 13/16 (81.3%) had NNRTI resistance impacting their prechange ART regimen. Furthermore, 1/16 (6.3%) had low-level dolutegravir resistance. In 3/16 (23.1%) 3, in 1/16 (6.3%) 2, in 5/16 (31.3%) 1, and in 7/16 (43.8%) 0 drugs of their prechange regimen were predicted to be active, corresponding to a median NNRTI susceptibility score of 0 (IQR: 0.0–0.1) and a median NRTI backbone susceptibility score of 1 (IQR: 0.4–1.4) (Figure 2). Four of 16 (25.0%) were predicted to have 2, 5/16 (31.3%) were predicted to have 1, and 7/16 (43.8%) were predicted to have no active NRTIs in their regimen after changing to dolutegravir.

### Detected Resistance ≥18 Months After Changing to Dolutegravir

Among the 85 participants, 8 (9.4%) had dolutegravir resistance (2/85 [2.4%] intermediate and 6/85 [7.1%] high-level) and 15/85 (17.6%) had NRTI resistance at the last time-point where a GRT was done. Among those with dolutegravir resistance, 1/8 (12.5%) had 2, 2/8 (25%) had 1, and 5/8 (62.5%) had 0 drugs predicted to be active in their postchange regimen, corresponding to a median dolutegravir susceptibility score of 0.0 (IQR: 0.0–0.1) and a median NRTI backbone susceptibility score of 0.5 (IQR: 0.0–1.0) (Figure 2). Among 5/8 individuals (62.5%) with dolutegravir resistance who had a GRT result before changing to dolutegravir, 1/5 (20.0%) had 1 and 4/5 (80.0%) had 0 active drugs in their prior NNRTI-containing regimen, corresponding to a median NRTI backbone susceptibility of 0.25 (IQR: 0.0–0.5). The remaining 3/8 without a prechange GRT result all had high-level resistance to their prior NNRTI core agent measured after changing to dolutegravir. Among those without dolutegravir resistance, the median susceptibility to the NRTI backbone was 1.0 (IQR: 0.75–2.0, n = 11) prechange and 2.0 (IQR: 2.0–2.0, n = 77) postchange to dolutegravir.

**Figure 3.**
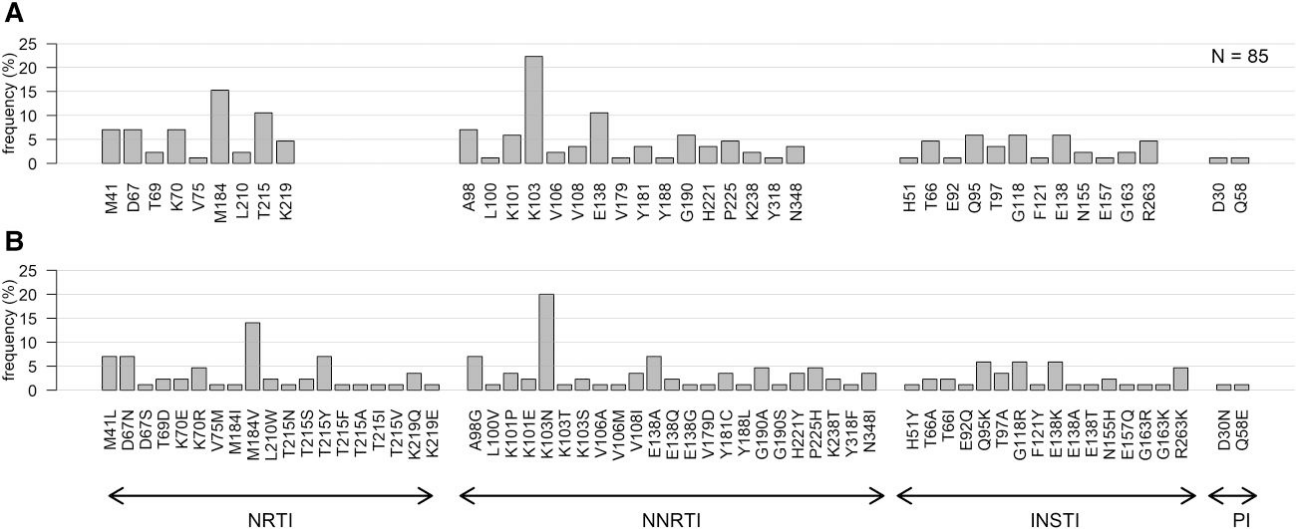
Resistance-associated mutations (identified at >5% variant frequency, with standalone effect according to Stanford HIV drug resistance database) at the last successfully sequenced sample while taking dolutegravir (N = 85). *A*, Frequency of participants with resistance-associated mutations at each position. *B*, Frequency of resistance-associated mutations (multiple per position and participant possible). INSTI, integrase strand transfer inhibitor; NNRTI, nonnucleoside transcriptase inhibitor; NRTI, nucleoside transcriptase inhibitor; PI, protease inhibitor; Four samples had a coverage of 1–10 reads on relevant NRTI/NNRTI resistance-associated mutations location and 2 samples had a coverage of 1–10 reads on relevant PI resistance-associated mutations location. For 1 sample, the reverse transcriptase was missing and for 3 samples, the protease was missing.

Figure 3 displays the frequency of detected mutations associated with resistance against NRTIs, NNRTIs, INSTIs, and protease inhibitors at each participant's last GRT. The most frequent resistance-associated mutations in the integrase region were Q95K, G118R, E138K, and R263K. Very few mutations associated with resistance to protease inhibitors were detected. One sample was HIV-1 subtype A1 and the others were subtype C.

### Characteristics of Participants With Dolutegravir Resistance

Table 2 shows characteristics of participants with successful GRT stratified by the detection of dolutegravir resistance. The last viral load ≤18 months before change to dolutegravir was ≥50 copies/mL for 6/8 (75.0%) participants with and 42/77 (54.5%) participants without dolutegravir resistance; this viral load was furthermore ≥100 000 copies/mL for 4/8 (50.0%) participants with and 4/77 (5.2%) participants without dolutegravir resistance. Table 3 displays the detailed clinical and social history of participants with dolutegravir resistance.

**Table 3.** Clinical and Social History of the 8 Participants With DTG Resistance

ID, Sex, Age at Change to DTG	Clinical History	Treatment History	Viral Loads^a^ (≤18 mo Before Change Until Data Closure; Copies/mL), Indicated As Time Since Change To DTG	Susceptibility Score to Core Agent	Susceptibility Score to NRTI Backbone	NRTI RAMs (Variant Frequency)^c^	NNRTI RAMs (Variant Frequency)^c^	INSTI RAMs (Variant Frequency)^c^	Reported Social History and Adherence Barriers
Participant 1 F, 27 y	CD4 nadir^a^: 700 cells/µL Time from diagnosis to ART start: 0 y Time taking ART at change to DTG: 6 y	*TDF-3TC-EFV*	*−12 mo: 200 000*	…	…	…	…	…	Lives alone History of taking ART only when health declines and defaulting from ART when health improves Treatment interruption lasting over 2 y while taking NNRTI-based ART Received adherence counselling for more than a year Transferred out
			*−8 mo: 2 000 000*	*EFV: 0.25* *DTG: 1.00*	*TDF-3TC: 1.00* *AZT-3TC: 1.00*	*M184V (100%)*	*Y181C (100.0%), H221Y (100.0%), N348I (88.3%), N348T (11.7%)*	*None*	
		AZT-3TC-DTG	5 mo: 200 000	…	…	…	…	…	
			10 mo: 2 000	…	…	…	…	…	
			13 mo: 1 000	DTG: 0.25	AZT-3TC: 1.00	M184V (99.2%)	K103R (99.0%), V179D (100.0%), Y181C (99.2%), Y188L (100.0%), H221Y (99.2%)	R263K (100.0%)	
			17 mo: 4 000	DTG: 0.25	AZT-3TC: 1.00	S68G (9.3%), M184V (100.0%)	K103R (97.8%), V179D (100.0%), Y181C (90.5%), Y188L (100.0%), H221Y (87.7%)	R263K (100.0%)	
			24 mo: 1 000	DTG: 0.25	AZT-3TC: 1.00 TDF-3TC: 1.00	M184V (99.9%)^b^	K103R (100.0%), V179D (99.4%), Y181C (99.5%), Y188L (99.9%), H221Y (100.0%)^b^	R263K (100.0%)^b^	
		TDF-3TC-DTG	26 mo: 20	…	…	…	…	…	
Participant 2 F, 27 y	CD4 nadir^a^: 700 cells/µL Time from diagnosis to ART start: 1 y Time taking ART at change to DTG: 7 y	*AZT-3TC-EFV*	*−18 mo: 17 000*	*EFV: 0.00* *DTG: 0.50*	*AZT-3TC: 0.50* *TDF-3TC: 1.00*	*K70R (99.0%), M184V (99.2%)^b^*	*A98G (100.0%), K103N (100.0%), P225H (100.0%)^b^*	*V157L (50.0%)* ^b^	History of viremia and adherence challenges Has received several sessions of enhanced adherence counselling Forgetfulness reported as main barrier to adherence History of condomless sex with partner with unknown HIV status (reported while taking EFV-based ART) Has epilepsy; was taking carbamazepine for epilepsy, but changed to sodium valproate >2 y after changing to DTG because of possible drug–drug interactions of carbamazepine with DTG
			*0 mo: 60*	…	…	…	…	…	
		TDF-3TC-DTG	7 mo: 700	DTG: 0.25	TDF-3TC: 0.50	D67N (44.7%), K70R (100.0%), M184V (100.0%), K219Q (100.0%)	A98G (100.0%), K103N (100.0%), P225H (100.0%)	G118R (100.0%)	
			19 mo: 10 000	DTG: 0.00	TDF-3TC: 0.50	D67N (40.7%), D67S (58.2%), K70R (100.0%), M184V (100.0%), K219Q (99.3%)	A98G (100.0%), K103N (100.0%), P225H (99.7%)	T66A (99.2%), G118R (100.0%), E138K (100.0%)	
			25 mo: 10 000	…	…	…	…	…	
			28 mo: 20 000	DTG: 0.00	TDF-3TC: 0.50	D67N (84.3%), D67S (15.7%), K70R (100.0%), M184V (100.0%), K219Q (100.0%)	A98G (100.0%), K103N (100.0%), P225H (100.0%)	T66A (99.0%), G118R (100.0%), E138K (100.0%)	
			42 mo: 3 000	DTG: 0.00	TDF-3TC: 0.50	D67N (79.0%), D67S (21.0%), K70R (99.6%), M184V (100.0%), K219Q (100.0%)	A98G (100.0%), K103N (96.1%), P225H (100.0%)	T66A (100.0%), G118R (100.0%), E138K (100.0%)	
			45 mo: 5 000	…	…	…	…	…	
Participant 3 F, 43 y	CD4 nadir^a^: 100 cells/µL Time from diagnosis to ART start: 2 y Time taking ART at change to DTG: 10 y	*TDF-3TC-EFV*	*−1 mo: 900 000*	*EFV: 0.00* *DTG: 1.00*	*TDF-3TC: 0.25*	*M41L (100.0%), E44D (13.9%), M184V (100.0%), L210W (44.7%), T215Y* (99.0%)	*K103N (100.0%), E138Q (100.0%)*	*None*	Has received several sessions of enhanced adherence counselling Main reported barriers to adherence include: lack of treatment supporter; use of traditional medicine to induce vomiting or diarrhea, believing this would eliminate HIV from the body; forgetting Reports condomless sex
		TDF-3TC-DTG	6 mo: < 20	…	…	…	…	…	
			17 mo: 4 000	DTG: 0.75	TDF-3TC: 0.50	M41L (92.1%), M184V (100.0%), T215Y (100.0%)	K103N (100.0%), E138Q (99.2%)	H51Y (99.8%)	
			28 mo: 10 000	DTG: 0.00	TDF-3TC: 0.50	M41L (97.0%), M184V (100.0%), T215Y (100.0%)	K103N (100.0%), E138Q (100.0%)	L74M (100.0%), G118R (100.0%), E138A (38.1%), E138K (61.8%), E157Q (11.7%), G163K (14.5%), G163Q (100.0%)	
			35 mo: 8 000	…	…	…	…	…	
			45 mo: 13 000	…	…	…	…	…	…
Participant 4 F, 26 y	CD4 nadir: (n.d.) Time from diagnosis to ART start: 0 y Time taking ART at change to DTG: 1 y	*TDF-3TC-EFV*	*−7 mo: < 20*	…	…	…	…	…	Lives with husband who also takes ART, with a recent documented VL <50 copies/mL Has received enhanced adherence counselling during various phases Forgetfulness reported as contributing factor to poor adherence Reports having 4 extramarital sexual partners, most of whom have an unknown HIV status (1 recently tested negative), and having condomless sex
		TDF-3TC-DTG	3 mo: 100	…	…	…	…	…	
			17 mo: 800	…	…	…	…	…	
			29 mo: 8 000	DTG: 0.00	TDF-3TC: 1.00	M41L (98.9%), M184V (98.1%)	A98G (99.2%), K103N (99.3%), P225H (85.8%)	T66I (46.1%), G118R (63.3%), E138 K (65.0%), R263K (44.0%)	
Participant 5 M, 9 y	CD4 nadir^a^: 800 cells/µL Time from diagnosis to ART start: (n.d.) Time taking ART at change to DTG: 6 y	*AZT-3TC-NVP*	*−2 mo: 30 000*	*NVP: 0.00* *DTG: 1.00*	*AZT-3TC: 0.00* *ABC-3TC: 0.25*	*E44D (19.9%), M184V (100.0%), L210W (27.0%), T215Y (100.0%)*	*A98G (22.6%), K101P (98.9%), K103S (99.8%)*	*None*	Lives in a child-headed household with 2 siblings (both minors) who act as treatment supporters, with occasional visits from a treatment supporter living far from their location Mother passed away No access to phone or similar devices for ART reminders; neighbor occasionally assists with timekeeping for ART For some periods, used traditional herbs to induce diarrhea, but was then advised against this by healthcare professionals Adherence challenges reported
		ABC-3TC-DTG^d^	8 mo: 10 000	DTG: 1.00	ABC-3TC: 0.50	M184V (99.3%), T215Y (99.4%)	K101P (98.7%), K103S (99.5%)	None	
			14 mo: 20 000	DTG: 0.75	ABC-3TC: 0.50	M184V (100.0%), T215H (5.2%), T215Y (90.9%)	K101P (99.4%), K103S (100.0%)	N155H (8.7%)	
			21 mo: 600	DTG: 0.00	ABC-3TC: 0.50	M184V (100.0%), T215H (40.0%),T215Y (60.0%)	K101P (100.0%), K103S (100.0%), Y318F (14.3%)	L74I (16.7), T66I (70.0%), G118R (100.0%), E138K (100.0%), R263K (42.9%)	
			26 mo: 100 000	DTG: 0.50	ABC-3TC: 2.00	None	K101P (97.3%), K103S (99.1%)	H51Y (39.2%), E92Q (17.4%)	
			34 mo: 10 000	DTG: 0.50	ABC-3TC: 0.50	M184V (100.0%), T215H (100.0%)	K101P (100.0%), K103S (100.0%)	H51Y (70.2%), E92Q (91.6%)	
			40 mo: missing	DTG: 0.25	ABC-3TC: 2.00	None	K101P (98.5%), K103S (99.4%)	H51Y (94.3%), E92Q (69.1%), T97A (5.3%), N155H (46.9%)	
			43 mo: 3 000	…	…	…	…	…	
Participant 6 M, 7 y	CD4 nadir^a^: 500 cells/µL Time from diagnosis to ART start: (n.d.) Time taking ART at change to DTG: 6 y	*AZT-3TC-NVP*	*−17 mo: 800 000*	…	…	…	…	…	Food insecurity; less adherent when food is not available due to concerns of side effects
		ABC-3TC-DTG	1 mo: 800	DTG: 1.00	ABC-3TC: 0.00	D67N (100.0%), T69D (98.1%), K70R (100.0%), M184V (100.0%), T215F (100.0%), K219Q (96.2%)	A98G (100.0%), K103N (100.0%), V108I (100.0%), Y181C (98.3%), G190A (100.0%), H221Y (100.0%)	None	
			7 mo: 100	…	…	…	…	…	
			30 mo: 2 000	DTG: 0.00	ABC-3TC: 0.00	D67N (100.0%), T69D (100.0%), K70R (100.0%), M184V (100.0%), T215F (100.0%), K219Q (100.0%)	A98G (100.0%), V108I (98.1%), Y181C (100.0%), H221Y (100.0%)	T66A (100.0%), T97A (9.1%), T97P (18.2%), G118R (100.0%), E138K (100.0%)	
			39 mo: 20 000	…	…	…	…	…	
			44 mo: 80 000	…	…	…	…	…	
Participant 7 M, 55 y	CD4 nadir^a^: 200 cells/µL Time from diagnosis to ART start: 0.0 y Time taking ART at change to DTG: 8.8 y	*TDF-3TC-EFV*	*−9 mo: 80 000*	…	…	…	…	…	Does not accept HIV status; mental health challenges (stress) in relation to HIV status documented Reports condomless sex Adherence challenges, reportedly due to lack of phone for reminders Unhealthy alcohol use reported Food insecurity; less adherent when food is not available due to concerns of side effects
			*−2 mo: 100 000*	*EFV: 0.00* *DTG: 1.00*	*TDF-3TC: 0.00* *AZT-3TC: 1.00*	*M41L (72.1%), K65R (74.7%), S68N (14.7%), K70N (5.9%), L74I (94.9%), M184V (100.0%), K219R (90.7%)*	*A98G (100.0%), K103N (100.0%), P225H (100.0%)*	*None*	
		AZT-3TC-DTG	11 mo: < 20	…	…	…	…	…	
			28 mo: 10 000	DTG: 0.00	AZT-3TC: 1.00	M41L (100.0%), M184V (100.0%)	A98G (100.0%), K103N (100.0%), P225H (100.0%)	T66I (85.4%), G118R (100.0%), E138K (100.0%), R263K (52.0%)	
			31 mo: 7 000	DTG: 0.00	AZT-3TC: 1.00	M41L (99.5%), M184V (100.0%)	A98G (100.0%), K103N (94.9%), K103S (5.1%), P225H (91.6%)	T66I (55.5%), G118R (89.0%), E138K (89.8%), R263K (37.6%)	
			37 mo: 20 000	…	…	…	…	…	
			38 mo: 30 000	DTG: 0.00	AZT-3TC: 0.00	M41L (99.1%), M184V (95.6%), T215Y (95.5%)	A98G (96.0%), K103N (96.5%), P225H (96.1%)	T66I (61.2%), G118R (100.0%), E138K (100.0%), R263K (87.7%)	
			42 mo: 40 000	…	…	…	…	…	
Participant 8 F, 34 y	CD4 nadir^a^: 300 cells/µL Time from diagnosis to ART start: 0.6 y Time taking ART at change to DTG: 10.5 y	*TDF-3TC-EFV*	*−15 mo: 30 000*	…	…	…	…	…	Unresolved family issues reported as negatively impacting adherence Reports condomless sex with partner Reports occasionally forgetting medication
			*−10 mo: < 20*	…	…	…	…	…	
		TDF-3TC-DTG	8 mo: 400	…	…	…	…	…	
			20 mo: < 20	…	…	…	…	…	
			23 m: 200	…	…	…	…	…	
			29 mo: < 20	…	…	…	…	…	
			35 mo: 40	…	…	…	…	…	
			37 mo: 20 000	DTG: 0.00	TDF-3TC: 0.00	M41L (27.3%), E44D (31.8%), D67N (53.5%), M184V (98.0%), L210W (80.4%), T215Y (100.0%)	K103N (90.7%), N348I (22.7%), N348M (77.3%)	N155H (26.2%), R263K (7.3%)	
			39 mo: < 20	…	…	…	…	…	

*Italic:* before change to DTG.

Abbreviations: 3TC, lamivudine; ABC, abacavir; ART, antiretroviral therapy; AZT, zidovudine; CD4, cluster of differentiation 4; DTG, dolutegravir; EFV, efavirenz; INSTI, integrase strand transfer inhibitor; NNRTI, nonnucleoside reverse transcriptase inhibitor; NRTI, nucleoside reverse transcriptase inhibitor; NVP, nevirapine; RAM, resistance-associated mutation; TDF, tenofovir disoproxil fumarate.

^a^Rounded to 1 significant figure.

^b^Minimal read depth below 10 at any resistance-associated mutation position.

^c^According to Stanford HIV drug resistance database, including resistance-associated mutations with a standalone and/or combinatory effect.

^d^Weight-based dosing with dispersible tablets.

## DISCUSSION

This study provides first insights into the dynamics and extent of emerging dolutegravir resistance to be expected in HIV programs. Among 157 participants with persistent or recurring viremia ≥18 months after changing to dolutegravir, 85 had a GRT result. Among these, 8 (9.4%) were found to have dolutegravir resistance, including 2 pediatric participants. Characteristics, clinical, therapeutic, and social history of the 8 participants with dolutegravir resistance were heterogeneous. However, the majority had a history of treatment failure on the previous NNRTI-based regimen with NRTI and NNRTI resistance, social circumstances favoring problems in treatment adherence, and all were in care at peripheral nurse-led health centers, as opposed to hospitals. However, in the overall cohort, 41.5% were in care at hospitals. Of note, among the 8 participants with dolutegravir resistance, the last viral load before changing to dolutegravir was ≥50 copies/mL in 6 and ≥100 000 copies/mL in 4 individuals.

The proportion of participants with dolutegravir resistance we observed is higher than what was reported in most (mostly shorter-term) previous observational studies including samples from Africa, namely in between 3% and 6% of persons with viremia [13, 18, 19]. The exceptions are 2 studies from Malawi. One study reported on 8 persons (30.0%) with at least intermediate-resistance to dolutegravir in the national program at least 7 months after change to (n = 7) or start of (n = 1) dolutegravir, albeit in a heavily preselected population [20]. The other reported 2 persons of 14 with viremia after change from NNRTI- to DTG-based ART with dolutegravir resistance, of whom both were viremic with NRTI backbone resistance at change to dolutegravir [11]. Outside of Africa, a high rate of major INSTI resistance-associated mutations was reported in an observational study from Brazil among people who first initiated on dolutegravir-based ART with no prior ART exposure, namely in 7 individuals (6.2%) taking dolutegravir for at least 6 months [31]. In line with previous reports, in our study all participants with dolutegravir resistance and prechange GRT had prior NRTI resistance [13, 16].

Three individual longitudinal resistance patterns in our study warrant particular consideration. Participant 1 achieved viral suppression <50 copies/mL 1 month after backbone change to TDF-3TC despite intermediate-level dolutegravir resistance and equal resistance level to the new regimen, which might reflect a period of increased adherence after changing to a more convenient 1-pill regimen. This appears plausible considering a recent case report describing 2 people taking 3TC-dolutegravir who resuppressed without treatment modification after identification of the R263K and M184V mutations [32]. In participant 5, repeated loss of resistance suggests longer periods of no drug pressure of the respective antiretrovirals. Indeed, fitness costs of mutations in the integrase region have been reported, inter alia, for E92Q [33], for G118R [34], and for R263K alone [30–32] or in combination with H51Y [35], E92Q [33], or G118R [34]. Furthermore, ABC-3TC-dolutegravir taken by this participant was not available as a fixed-dose combination at the time of the study. Periods of increasing dolutegravir resistance with loss of backbone resistance suggest that this individual may, for certain periods, have taken dolutegravir monotherapy. Participant 8 repeatedly had full viral suppression despite reported high-level resistance to all drugs in her regimen. This suggests that many of the detected mutations that, combined, confer high-level resistance to dolutegravir (N155H [observed variant frequency: 26.2%], R263K [7.3%]) and TDF (M41L [27.3%], E44D [31.8%], D67N [53.5%], L210W [80.4%], and T215Y [100.0%]) might not be present on the same HIV genomes.

Although overall dolutegravir resistance is still considered rare in Africa, acquired and transmitted dolutegravir resistant is an emerging threat [11, 13, 18–20]. This is underlined by the recent first report of dolutegravir resistance in a treatment-naïve infant with perinatal acquisition of HIV [36]. Of note, the relatively early emergence of dolutegravir resistance in our study calls into question current guidelines negating the need for resistance testing within 24 months of initiating dolutegravir-based ART [14, 15]. Given the limited laboratory capacity and high costs associated with GRT, research should focus on developing criteria to predict the likelihood of resistance and narrow down the patient population requiring GRT.

This study is not without limitations. First, our results are limited to participants who changed from NNRTI- to dolutegravir-based ART. They can thus not be extrapolated to participants who started on dolutegravir-based ART. Second, we included GRT results with a relatively low read coverage and with incomplete coverage outside the integrase region, which could have limited sensitivity for resistance-associated mutations with a low variant frequency in this subset of samples. Third, we did not consider whether resistance-associated mutations measured with a low variant frequency were located on the same HIV genomes or whether the role of mutations associated with INSTI resistance were located outside the integrase region [37]. Fourth, GRT was only available for roughly half of the individuals fulfilling the virologic criteria; however, where GRT was missing, this was mostly due to not reaching participants for informed consent or unavailability of a sample for sequencing. Finally, numbers were too low to draw clear conclusions regarding correlates of emergent dolutegravir resistance.

In conclusion, our findings reaffirm previously reported high viral suppression rates with dolutegravir in routine African HIV programs. In the VICONEL cohort, the prevalence of dolutegravir resistance among people who changed from NNRTI- to dolutegravir-based ART was approximately 1 per 1000. Nevertheless, the prevalence of dolutegravir resistance among those with persistent or recurring viremia after changing to dolutegravir is concerning. Immediate and appropriate measures must be taken to sustain the advancement achieved through the rollout of dolutegravir in Africa. This should include monitoring for emergent dolutegravir resistance in HIV programs, as well as guaranteeing timely diagnosis of dolutegravir resistance at the patient level with access to effective, well-tolerated alternative regimens.

## Notes

***Data sharing*.** Deidentified individual participant data will be made available upon reasonable request to the corresponding author.

***Financial support***. This study was funded by the Swiss National Science Foundation (Eccellenza grant number PCEFP3_181355 and R4D grant number IZ07Z0_160876/1 to N. D. L.), and the Research Fund of the University of Basel (grant number 3ZX1404 to N. T.). J. A. B. receives her salary through grants from Fondation Botnar (grant number REG-19-008 to J. A. B. and N. D. L.) and the Research Fund of the University of Basel (grant 3ZX1422 to J. A. B.). The funders had no role in study design, data collection, data analysis or interpretation, the writing of the report, or the decision to submit the paper for publication.
